# Regional lung deflation with increased airway volume underlies the functional response to bronchodilators in chronic obstructive pulmonary disease

**DOI:** 10.14814/phy2.14330

**Published:** 2019-12-26

**Authors:** Naoya Tanabe, Susumu Sato, Shigeo Muro, Hiroshi Shima, Tsuyoshi Oguma, Kazuya Tanimura, Atsuyasu Sato, Toyohiro Hirai

**Affiliations:** ^1^ Department of Respiratory Medicine Graduate School of Medicine Kyoto University Kyoto Japan; ^2^ Department of Respiratory Medicine Nara Medical University Nara Japan

**Keywords:** airway, bronchodilator, COPD, CT, hyperinflation, total airway count

## Abstract

Bronchodilators, including long‐acting muscarinic antagonists (LAMAs), improve airflow limitation and lung hyperinflation in patients with chronic obstructive pulmonary disease (COPD). While bronchodilators increase airway caliber and deflate the lungs, little is known about the effects of the local interaction between airway dilation and lung deflation on functional improvements resulting from bronchodilator therapy. This study aimed to explore whether lung deflation with increased airway volume in the upper and lower lung regions would produce different physiological responses to LAMA therapy. Using the clinical data of 41 patients with COPD who underwent spirometry and inspiratory computed tomography (CT) before and 1 year after LAMA treatment, we measured the 1‐year change in the airway tree to lung volume percentage ratio (AWV%) for the right upper, middle, and lower lobes (RUL, RML, and RLL) and the left upper and lower lobes (LUL and LLL), and total airway count (TAC) identifiable on CT in relation to the forced expiratory volume in 1 s (FEV_1_). The results showed that LAMA treatment significantly increased the FEV_1_ and AWV% of the RUL, RML, RLL, LUL, and LLL. Increased AWV% in the RLL and LLL, but not in the RUL and LUL, was correlated with increased FEV_1_. In the multivariate analysis, the increased AWV% in the RLL was associated with the increased FEV_1_ independent of the change in TAC in the RLL after treatment. This is the first study to show that the physiological improvements after bronchodilator treatment in COPD could be mainly due to the combination of regional deflation and increased airway volume of the lower lobes.

## Graphical abstract

Bronchodilators improve airflow limitation and lung hyperinflation in patients with chronic obstructive pulmonary disease (COPD) by increasing airway calibre and deflate the lungs, but little is known about effects of the local interaction between airway dilation and lung deflation on functional improvements resulting from bronchodilator therapy. This study showed that the improved airflow limitation after long‐acting bronchodilator treatment was significantly correlated with an increase in the airway tree to lung volume percentage ratio in the lower lobe, but not in the upper lobes. These data suggest that the physiological improvements after bronchodilator treatment in COPD could be mainly due to the combination of regional deflation and increased airway volume of the lower lobes.

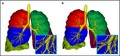

## 1 INTRODUCTION

Chronic obstructive pulmonary disease (COPD) is characterized by chronic airflow limitation and lung hyperinflation induced by a combination of increased airway resistance due to luminal narrowing and decreased elastic recoil of the parenchyma due to emphysema (Vogelmeier et al., 2017). These pathophysiological changes are closely associated with symptoms, impaired quality of life, and poor prognosis (Casanova et al., 2005; O'Donnell, Elbehairy, Webb, & Neder, 2017). While no treatment is currently available for emphysema, bronchodilators such as long‐acting muscarinic antagonists (LAMAs) and long‐acting beta‐agonists (LABAs) can partially relieve airflow limitation and symptoms in patients with COPD (Donohue et al., 2010; Tashkin et al., 2008). Moreover, as a consequence of the direct dilating effect on the airway lumen, the bronchodilators facilitate emptying of the lung at expiration and achieve a reduction in lung hyperinflation (lung deflation) (Casaburi et al., 2014; Hohlfeld et al., 2018). Therefore, bronchodilators have been recently considered as “lung deflators” (Kostikas & Siafakas, 2016).

The pulmonary function test is the gold standard for evaluating bronchodilator response (Tashkin et al., 2008; Vestbo et al., 2017). Many clinical studies have used a change in the forced expiratory volume in 1 s (FEV_1_) on spirometry as the main outcome because spirometry is noninvasive, readily accessible, and highly reproducible. Other measurements regarding lung hyperinflation, such as residual volume (RV), functional residual capacity (FRC), and ratio of RV to total lung capacity (TLC), are also used to detect lung deflation (Hohlfeld et al., 2018; Kostikas & Siafakas, 2016). However, these physiological measures assess only the entire lung, including both the airway and lung. The underlying local structural responses to bronchodilators are still not fully understood.

Chest computed tomography (CT) is a common imaging technique used to analyze the local structures that pulmonary function tests have difficulties in detecting (Hackx et al., 2015; Hasegawa et al., 2009; Hoshino & Ohtawa, 2014; Shimizu et al., 2015, 2016; Tanabe, Muro, Oguma, et al., 2012; Yasui et al., 2013). For example, the heterogeneity of emphysema between the upper and lower regions of the lung affects pulmonary function independently of the whole‐lung emphysema severity (Boueiz et al., 2018; Fuseya et al., 2018; Ju et al., 2014; Nakano et al., 1999; Tanabe, Muro, Tanaka, et al., 2012). In the assessment of bronchodilator responses, CT measurements can reveal regional changes in the airway lumen diameter (Shimizu et al., 2015) and pharmacological volume reductions of the entire lung and within lung lobes (De Backer et al., 2012; Tanabe, Muro, Oguma, et al., 2012; Vos et al., 2016).

In healthy persons, the airway‐parenchyma interdependence allows airway caliber to be increased when the lung is inflated. However, this bronchodilation at inspiration is diminished in patients with COPD (Diaz et al., 2012; Scichilone et al., 2004). A recent CT study enabled an assessment of the interaction between the airway and parenchyma by measuring the airway tree to lung volume ratio on CT, termed the AWV%, and showed that a reduction in the AWV% is closely associated with a decrease in FEV_1_, independent of emphysema and the dimension of the central airways in COPD (Tanabe et al., 2019). Therefore, local lung deflation accompanied by an increase in corresponding airway volume could potentially and reasonably account for the improvement in FEV_1_ after bronchodilator therapy in patients with COPD.

This study aimed to investigate whether increases in the AWV% in the right upper, middle, and lower lobes (RUL, RML, and RLL) and the left upper and lower lobes (LUL and LLL) had different associations with the change in FEV_1_ that occurs in response to standard LAMA treatment by using the clinical data of a prospective cohort, including patients with COPD who underwent CT scans before and 1 year after LAMA treatment.

## 2 MATERIALS AND METHODS

### 2.1 Study design

This study retrospectively analyzed the data of a prospective observational study conducted at Kyoto University (Tanabe et al., 2011; Terada et al., 2008). The study was performed in accordance with the Declaration of Helsinki and was approved by the Ethics Committee of Kyoto University (approval No. E182 and R0311‐2). Written informed consent was obtained from all participants.

The inclusion criteria were as follows: (a) smoking history of at least 20 pack‐years, (b) a diagnosis of COPD confirmed by postbronchodilator FEV_1_/forced vital capacity (FVC) ratio <70% (Vogelmeier et al., 2017), and (c) no history of lung resection surgery or other lung diseases, such as bronchial asthma or interstitial lung disease. Among all participants, the present analyses included the patients who underwent chest inspiratory CT scans at TLC and pulmonary function tests before and 1 year after starting tiotropium treatment between 2006 and 2015. We excluded patients who received LAMAs and/or LABAs before starting tiotropium and those whose medications for lung disease were changed during the 1‐year interval between the two scans.

Postbronchodilator spirometry was performed 6 months and 1 year after starting tiotropium treatment using a Chestac‐65V device (Chest MI Corp.). Lung volume evaluations and diffusion capacity measurements were also performed before treatment. Chest CT scans with a 0.5 mm slice thickness were acquired with a peak kilovoltage of 120, a 0.5 s exposure time, and auto‐exposure control using an Aquilion 64 scanner (Cannon Medical). Reconstruction was performed with a high spatial frequency algorithm (FC56).

### 2.2 CT measurements

**Figure 1:**
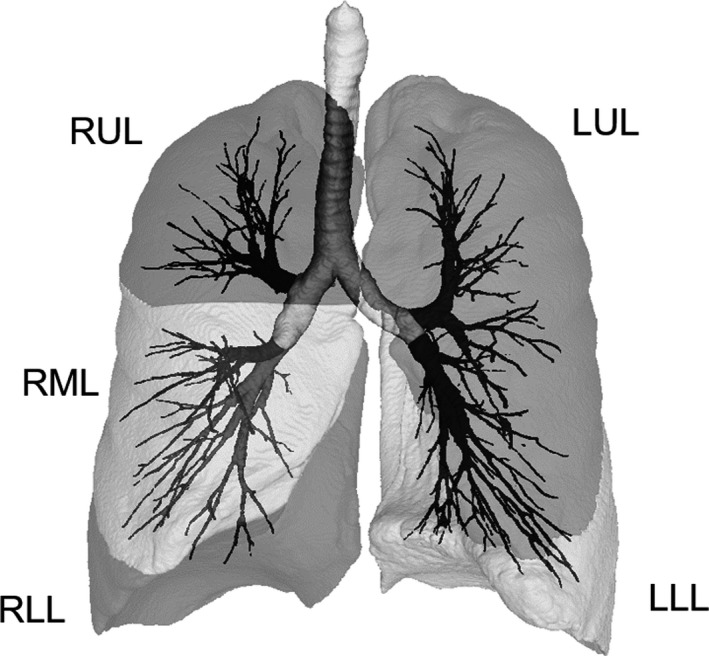
Methods used to calculate the airway tree to lung percentage ratio using CT. The airway tree and each lobe were segmented, and the volumes of tree portions in the right upper, middle, and lower lobes (RUL, RML, and RLL) and the left upper and lower lobes (LUL and LLL) were measured together with the lung volumes of the RUL, RML, RLL, LUL, and LLL. The airway tree to lung volume percentage ratio (AWV%) for each region was then calculated

The details of the calculation method for AWV% were recently reported (Tanabe et al., 2019). Briefly, the segmentation of the airway tree was automatically generated without any manual editing using a SYNAPSE VINCENT volume analyser (FUJIFILM Medical). The segmented airway trees were further segmented into portions of the RUL, RML, RLL, LUL, and LLL with ITK‐SNAP software (Yushkevich et al., 2006). Lobe segmentation was also performed with manual correction when necessary, and then the volumes of the RUL, RML, RLL, LUL, and LLL were measured (Figure 1). The AWV% values for the RUL, RML, RLL, LUL, and LLL were calculated as a percentage ratio of the airway volume in the RUL, RML, RLL, LUL, and LLL to the volume of the RUL, RML, RLL, LUL, and LLL, respectively, using custom‐made software. To assess the number of airway branches identifiable on CT, the total airway count (TAC) was separately measured using the centerline of the segmented airway trees in the RUL and RLL (Tanabe et al., 2019). The percentage ratio of voxels <−950 HU to the lung voxels, namely, the low attenuation volume percent (LAV%), was also measured for each lobe to assess emphysematous changes on CT.

### 2.3 Statistics

The data are expressed as the mean ± standard deviation (*SD*). Paired *t*‐tests were used to compare the CT measurements and pulmonary function test results before and after LAMA treatment. Spearman rank correlation analysis was used to evaluate the associations between changes in CT measurements and FEV_1_. Statistical analysis was performed with R Core Team (2015). A *p* value <.05 was considered statistically significant.

## 3 RESULTS

**Figure 2:**
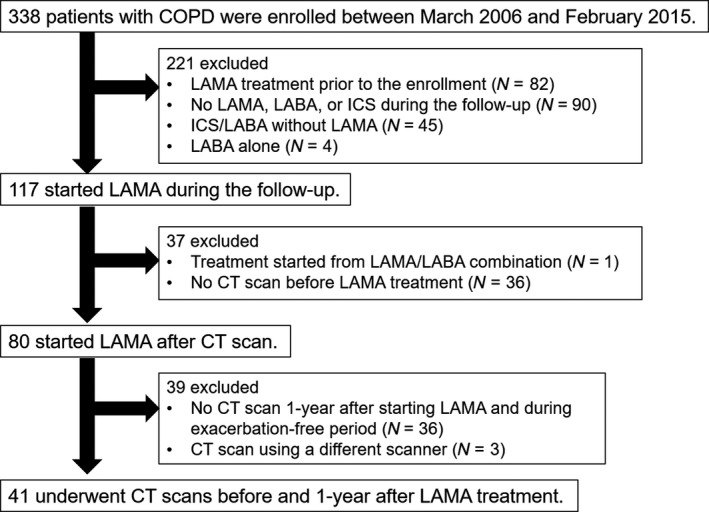
Patient flowchart. ICS, inhaled corticosteroid; LABA, long‐acting β agonist; LAMA, long‐acting muscarinic antagonist

As shown in the CONSORT diagram (Figure 2), 338 patients with COPD were enrolled in the Kyoto University cohort study from January 2006 to February 2015. Among these patients, 80 patients underwent inspiratory CT scans before starting LAMA treatment (tiotropium). After excluding 36 patients who did not undergo the follow‐up CT scan 1 year after starting LAMA treatment and 3 patients who underwent follow‐up CT scans with a different scanner than the Aquilion 64 scanner, a total 41 patients were included in this study. Table 1 summarizes the demographics of these patients. D_LCO_ (% predicted) was 49.2 ± 18.7%, and LAV% for the entire lung was 31.4 ± 8.7%. In the lobar assessment, LAV% in the RUL was greater than in the RLL (32.7 ± 9.9 and 29.1 ± 9.6%, *p* = .005), and LAV% in the LUL was greater than in the LLL (32.8 ± 9.2 and 29.6 ± 10.0%, *p* = .002).

**Table 1:** Patient demographics (*n* = 41)

Age	71.4 ± 7.1
Sex (male:female)	39:2
Height (cm)	163.0 ± 6.8
Weight (kg)	59.1 ± 9.0
Smoking status (current:former)	8:33
Pack‐years	62.0 ± 34.7
FEV_1_ (ml)	1,345 ± 476
FEV_1_ (% predicted)	51.0 ± 16.8
FEV_1_/FVC (%)	44.3 ± 11.6
FRC (% predicted)	107.7 ± 17.5
RV/TLC (%)	44.7 ± 9.8
D_LCO_ (% predicted)	49.2 ± 18.7
LAV% (%)	31.4 ± 8.7

Note

Data are expressed as the mean ± *SD*.

Abbreviations: D_LCO_, diffusion capacity of the lung for carbon monoxide; FEV_1_, forced expiratory volume in 1 s; FRC, functional residual capacity; FVC, forced vital capacity; LAV%, percent low attenuation area less than −950 Hounsfield units (HU) on CT; RV/TLC, residual volume/total lung capacity.

**Figure 3:**
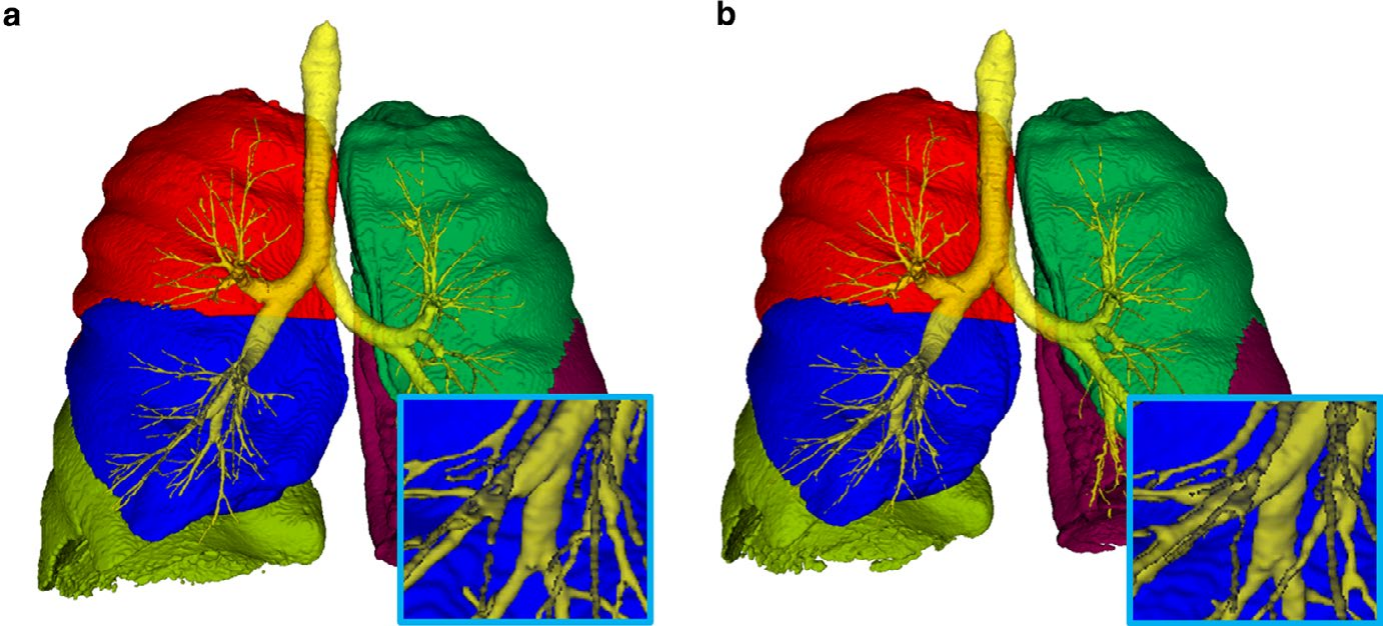
Comparison of airway and lung volumes before and 1 year after bronchodilator treatment. The airway tree and lung volumes were compared before (a) and 1 year after (b) starting long‐acting muscarinic antagonist. Red, blue, light green, dark green, and dark red indicate the right upper, middle, and lower lobes, and the left upper and lower lobes, respectively

**Figure 4:**
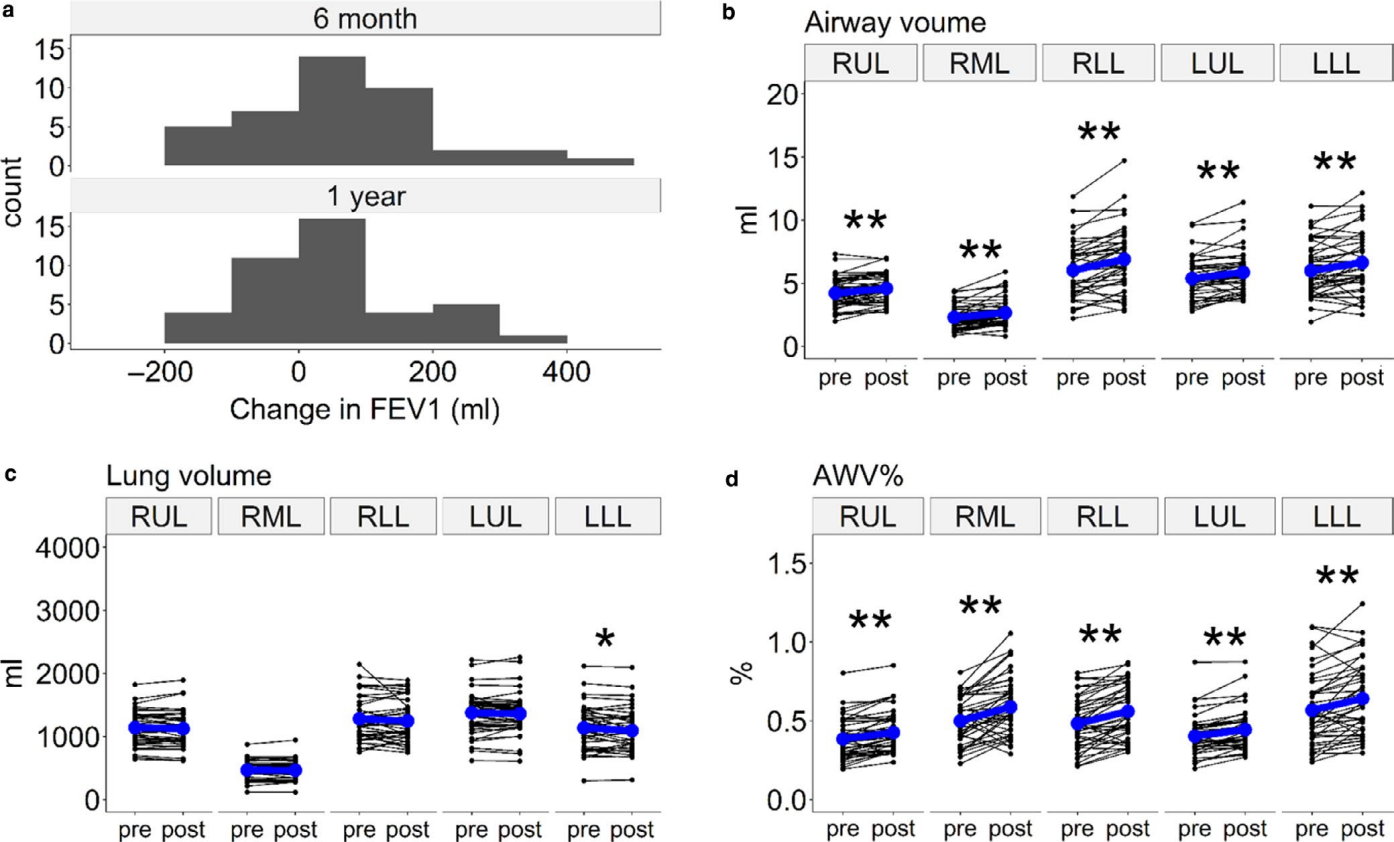
Changes in lung function and CT measures after bronchodilator treatment. (a) A histogram of the 6‐month and 1‐year changes in forced expiratory volume in one second (FEV_1_) after starting long‐acting muscarinic antagonist (LAMA) treatment. (b) Airway volume, (c) lung volume, and (d) airway tree to lung volume percentage ratio (AWV%) in the right upper, middle, and lower lobes (RUL, RML, and RLL) and the left upper and lower lobes (LUL and LLL). * and ** indicate *p* < .05 and *p* < .01, respectively

Figure 3 shows an example case that visualizes an increase in airway volume and a decrease in lung volume, thus leading to a decrease in AWV%. In Figure 4, the majority of the patients showed increases in FEV_1_ 6 months and 1 year after starting LAMA treatment. These increases in FEV_1_ 6 months and 1 year after treatment were closely associated with each other (rho = 0.59, *p* < .01). The airway volumes and AWV% of the RUL, RML, RLL, LUL, and LLL were significantly increased, and the lung volume of the LLL, but not of the RUL, RML, RLL, or LUL, was significantly decreased after LAMA treatment.

**Figure 5:**
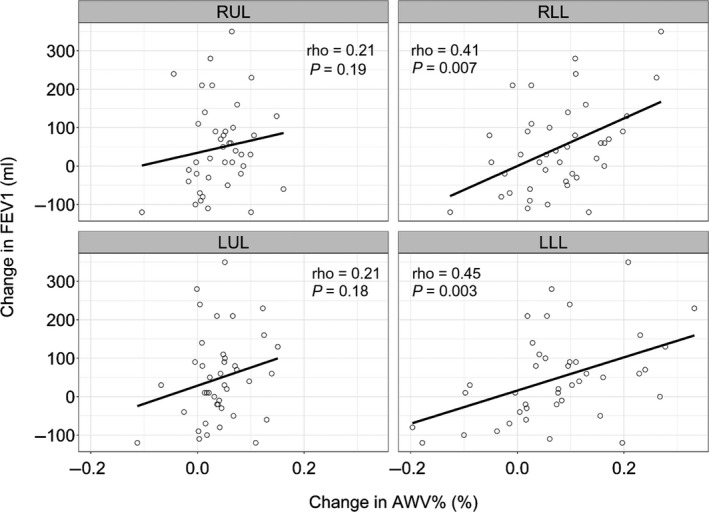
Associations between local changes in the airway volume percentage and lung function after bronchodilator treatment. The changes in the airway tree to lung volume percentage ratio (AWV%) in the right and left lower lobes (RLL and LLL), but not in the right and left upper lobes (RUL and LUL), showed a significant correlation with the forced expiratory volume in one second (FEV_1_) 1 year after long‐acting muscarinic antagonist (LAMA) treatment

**Figure 6:**
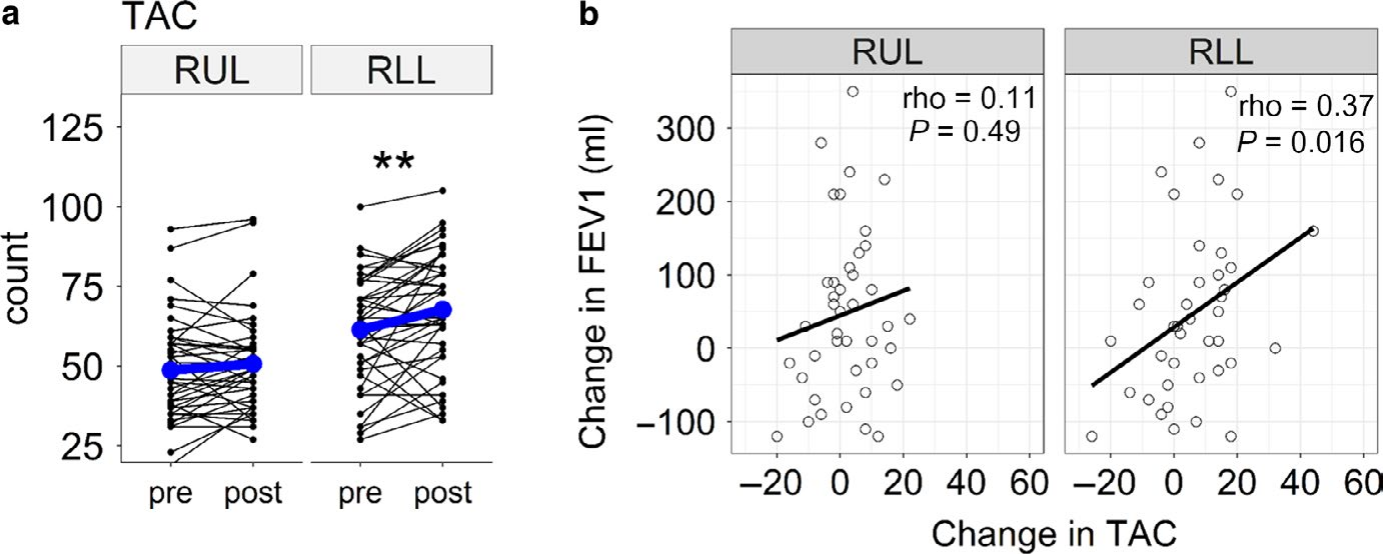
Change in total airway count and lung function after bronchodilator treatment. (a) Total airway count (TAC) in the right lower lobe (RLL), but not in the right upper lobe (RUL), increased after bronchodilator treatment. (b) The change in TAC in the RLL, but not in the RUL, was significantly associated with the change in the forced expiratory volume in one second (FEV_1_) 1 year after long‐acting muscarinic antagonist (LAMA) treatment

Table 2 summarizes the correlations between the changes in the CT indexes and FEV_1_ after LAMA treatment. The increases in the AWV% in the RLL and LLL (but not in the RUL and LUL) were significantly associated with improvements in FEV_1_ (Figure 5). In Figure 6, TAC in the RLL, but not in the RUL, was increased after LAMA treatment. The increase in TAC in the RLL was associated with the improvement in FEV_1_. In the multivariate linear regression analysis (Table 3), the increase in the AWV% in the RLL was significantly associated with the change in FEV_1_ after adjusting for the change in TAC and the baseline emphysema severity on CT, as assessed as LAV%.

**Table 2:** Correlations between the changes in CT measurements and FEV_1_ after bronchodilator treatment

	Region	Rho	95% CI	*p* value
Change in lung volume	RUL	−0.20	−0.48 to 0.11	.20
	RML	−0.25	−0.52 to 0.06	.11
	RLL	−0.30	−0.56 to 0.01	.06
	LUL	−0.35	−0.60 to −0.05	.02
	LLL	−0.14	−0.43 to 0.18	.40
Change in airway volume	RUL	0.17	−0.15 to 0.45	.29
	RML	0.16	−0.16 to 0.45	.32
	RLL	0.22	−0.10 to 0.49	.18
	LUL	0.09	−0.22 to 0.39	.57
	LLL	0.31	−0.0001 to 0.56	.05
Change in AWV%	RUL	0.21	−0.10 to 0.49	.19
	RML	0.23	−0.09 to 0.50	.15
	RLL	0.41	0.12 to 0.64	.007
	LUL	0.21	−0.10 to 0.49	.18
	LLL	0.45	0.17 to 0.67	.003

Note

The rho values are Spearman rank correlation coefficients.

Abbreviations: 95% CI, 95% confidence interval; AWV%, airway tree to lung volume percentage ratio.; left upper lobe; LLL, left lower lobe; RLL, right lower lobe. LUL; RML, right middle lobe; RUL, right upper lobe.

**Table 3:** Multivariate linear regression analysis of the associations between CT measurements and changes in FEV_1_ after bronchodilator treatment

	Variables	Standardized estimate	95% CI	*p* value
RUL	Change in AWV%	−0.07	−0.31 to 0.46	.70
	Change in TAC	0.07	−0.31 to 0.45	.71
	Baseline LAV%	0.13	−0.21 to 0.48	.43
RLL	Change in AWV%	0.35	0.03 to 0.67	.03
	Change in TAC	0.16	−0.16 to 0.48	.33
	Baseline LAV%	0.19	−0.10 to 0.48	.19

Abbreviations: 95% CI, 95% confidence interval; AWV%, airway tree to lung volume percentage ratio; LAV%, low attenuation volume percent; TAC, total airway count.

## 4 DISCUSSION

This study showed that LAMA treatment increased the FEV_1_ and AWV% in the RUL, RML, RLL, LUL, and LLL in patients with COPD. Importantly, the results showed that the increased FEV_1_ was associated with an increased AWV% in the RLL and LLL, but not in the RUL, RML, and LUL. To the best of our knowledge, this is the first study to show that regional lung deflation with an increased airway volume in the lower lung lobes could account for the improvements in FEV_1_ after bronchodilator therapy.

Earlier studies have shown that dilation of the airway lumen and deflation of the lung parenchyma are essential for achieving a satisfactory response to bronchodilators (Hasegawa et al., 2009; Hohlfeld et al., 2018; O'Donnell et al., 2017). However, very few studies have focused on the interaction between these structures and bronchodilators. Thus, the present data extended upon the previous findings by showing that an increase in AWV% in the RLL and LLL on CT is associated with an increase in FEV_1_ after LAMA treatment.

The present data also showed that TAC in the RLL increased after LAMA treatment, and this increased TAC was associated with the increased FEV_1_. TAC has been recently shown to reflect the pathology of the small airway disease in COPD (Kirby et al., 2019), to be decreased even in mild COPD and to predict future lung function decline (Kirby et al., 2018). However, as far as we are aware, no report has examined the effects of bronchodilators on TAC. Therefore, a future study should investigate whether the increase in TAC after bronchodilator treatment is associated with the clinical outcomes of patients with COPD.

Notably, the multivariate analysis showed that the increase in AWV% was associated with the improvement in FEV1 independently of the changes in TAC. A previous study showed that while AWV% reflects the dimension of the central airways and TAC on CT, AWV% is associated with airflow limitation and gas trapping independent of emphysema, the dimension of the central airway, and TAC (Tanabe et al., 2019); furthermore, the study suggested that in addition to each structural change in the airway and lung, the interaction between airway and lung volumes would affect pulmonary function. Taken together, the present results suggest that LAMAs work by increasing the lumen diameter of the airways identifiable on the baseline CT scan and the number of airways in the lower lobe identifiable on the follow‐up CT scan, which could reverse the ratio of the disproportionately small airway lumen volumes to the relatively large lung and might mechanistically improve breathing efficiency.

The finding that the associations of increased AWV% with FEV_1_ change are found only in the lower lobes is of great interest. The present data also showed that while all the airway volumes in the RUL, RML, RLL, LUL, and LLL were increased after LAMA treatment, the increased airway volumes in the LLL tended to be associated with the increase in FEV_1_. Because the lower lobes have greater compliance than the upper lobes, a higher degree of deflation might occur in the lower lobe more frequently than in the upper lobe. Indeed, regional ventilation is greater in the lower region than in the upper region in healthy subjects (Ball, Stewart, Newsham, & Bates, 1962), and the extent of emphysema in the lower lobe is more closely associated with airflow limitations than the extent in the upper lobe (Labaki et al., 2019). These previous findings suggest that the lower lung lobes could contribute more strongly to expiratory airflow than the upper lung lobes do. Moreover, the patients in this study showed greater LAV% in the upper lobes than in the lower lobes, indicating that nonemphysematous regions are more distributed in the lower lobes. Therefore, we postulate that bronchodilators improve airflow limitation mainly by increasing airway lumen sizes and facilitating ventilation of the lower lung lobes.

Another advantage of this study is the visualization of the bronchodilator effect. We believe that the increased visibility of the airway tree due to the increased AWV% has a direct impact for patients who would use LAMAs, and this state‐of‐the‐art visualization may help increase patient adherence to long‐acting bronchodilators, especially patients with mild COPD. Lung function declines more in mild COPD than in severe COPD (Tantucci & Modina, 2012), and an irreversible loss of the terminal bronchioles even occurs in mild stages of COPD (Koo et al., 2018); however, a therapeutic intervention with tiotropium relieved the annual decline in FEV_1_ for these mild COPD patients (Zhou et al., 2017). Therefore, encouraging patients with mild COPD to appropriately adhere to long‐acting bronchodilators is clinically important.

We recognize that the small sample size is a limitation of this study. Unlike other studies that compared the natural course of the disease, this study investigated the effects of bronchodilators on the imaging features, which cannot be evaluated in a larger scale study because CT scanners are not as easily accessible for many patients. However, the use of a single scanner with fixed scanning conditions is a great advantage of this study, and the present results successfully revealed the association of an increased AWV% in the RLL and LLL with the improvement in FEV_1_. Although significant changes in AWV% were detected on the follow‐up scans obtained 1 year after LAMA treatment, the change in FEV_1_ after 1 year of treatment was closely associated with that after 6 months of treatment. Thus, it is possible that LAMA treatment consistently has the same effect on airway and lung volumes on any given day after starting treatment.

In conclusion, this study showed that the improvements in FEV_1_ resulting from LAMA treatment for COPD could be mainly due to the combination of increased airway volume and local deflation of the lower lung lobes. The finding definitely stimulates interest in further research to investigate whether patients with hyperinflation of the lower lobes might respond better to LAMA treatment than those without, and LAMAs should reach the lower lobes to sufficiently facilitate lung deflation and improve the FEV_1_ in patients with COPD.

## CONFLICT OF INTEREST

This work was partially supported by a grant from FUJIFILM Medical for data analysis.

## AUTHOR CONTRIBUTIONS

N. T, S. S, S. M, H.S., T.O., K.T., and A.S. contributed to collecting, analyzing, and interpreting the data. N.T., S.S., S.M., and T. H contributed to conceiving and designing the study as well as delineating the hypothesis. N.T., S. S, S.M., and T.H. contributed to writing the manuscript.
